# Establishment of a reliable transfer process for fabricating chemical vapor deposition-grown graphene films with advanced and repeatable electrical properties

**DOI:** 10.1039/c8ra02478b

**Published:** 2018-05-30

**Authors:** Dongyun Sun, Wei Wang, Zhaoping Liu

**Affiliations:** Ningbo Institute of Materials Technology and Engineering, Chinese Academy of Sciences Ningbo Zhejiang 315201 P. R. China wangwei@nimte.ac.cn liuzp@nimte.ac.cn; Nano Science and Technology Institute, University of Science and Technology of China China

## Abstract

Graphene films grown by the chemical vapor deposition (CVD) method have attracted intensive attention due to their native advantages of both high quality and large quantity for commercial applications. However, previously reported graphene films have exhibited uncertain and conflicted electrical properties that greatly hinder them from being used to build reliable electrical devices because of incompatibility during the complex and multifarious transfer process. Herein, the relationship between the transfer parameters and electrical performance was systematically studied. It demonstrates that cracking during the transfer process causes significant loss of carrier mobility and hence an increase in sheet resistance. Additionally, unstable doping plays a key role in the carrier density and hence greatly influences the sheet resistance. By introducing HCl as a doping agent, graphene films with repeated sheet resistance of approximately 300 ohm sq^−1^ can be realized. This work establishes a facile and reliable route to fabricate graphene films with advanced and repeatable electrical properties, which is significant and essential for fair evaluation of CVD-grown graphene films and further practical applications.

## Abstract 2

Graphene films grown by the chemical vapor deposition (CVD) method have attracted intensive attention due to their native advantages of both high quality and large quantity for commercial applications.

## Graphical abstract

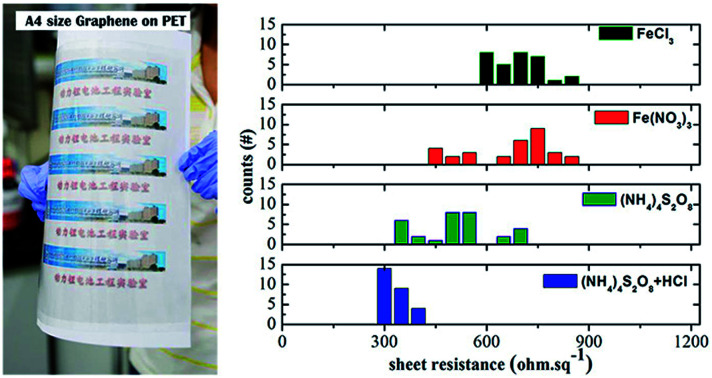

## 1. Introduction

Recently, there has been great interest in graphene because of its impressive mechanical and physical properties.^1–4^ Among various fabrication approaches of graphene films, the chemical vapor deposition (CVD) method has been considered to be one of the most important approaches because graphene films of both high quality and large quantity are obtained by its use.^5–7^ It has been predicted that CVD-grown graphene films will play a significant role in the operation of the optoelectronic, electrical, and flexible devices that are anticipated for the future.^8,9^ It is known that before proceeding to practical devices, CVD-grown graphene films must be transferred from catalyst substrates to desirable substrates.^10,11^ As a typical example, monolayer graphene grown on a Cu substrate has been widely studied and transferred to polyethylene terephthalate (PET) substrate that can be used as flexible transparent conducting films for next-generation flexible electronics.^12,13^ However, this process usually leads to an unavoidable decrease in electrical performance due to uncertain cracks formation and pollution problems during the process.^14–16^

Various transfer methods have been developed to solve those problems, including the polymer-assisted transfer method,^17,18^ the thermal release tape (TRT) method,^19–21^ and the glue-assisted method.^22,23^ Earlier methods involved growing graphene film on a Ni substrate and transferring it to a Si substrate by employing polydimethylsiloxane (PDMS) as an intermediate support layer.^24^ Polymethyl methacrylate (PMMA) has subsequently been widely accepted and used instead of PDMS to transfer graphene films and other ultrathin films, because it can be easily removed by solvents while maintaining clean and complete samples.^25^ However, the size of the resultant films in these approaches is usually limited to a few centimeters, which is not sufficiently large to be used as a next-generation transparent conducting films for building practical devices. It was firstly reported by Samsung that large scale graphene films with sizes up to 30 inches were successfully fabricated using thermal release tape as an intermediate transfer layer, which made it possible to build an operable and useful touch panel.^20^ This impressive progress had attracted broad attention, but the difficulty of operation led to some conflicting results in the reports that followed. Pressure-sensitive adhesive films and silicone films have been used as improved intermediate layers to produce graphene films that exhibit more advanced completeness and electrical properties.^26^ Moreover, Sony Corporation produced a 100 m-long high-quality graphene transparent conductive films by roll-to-roll CVD and transfer with the assistance of UV-glue bonding.^23^ Undoubtedly, the correct selection of the transfer method plays a key role in determining the final device performance.^11,27–29^

In addition to the numerous transfer methods, another non-negligible and irreplaceable function comes from the etchant for removing catalyst substrates, which usually contains iron chloride (FeCl_3_),^30^ iron nitrate (Fe(NO_3_)_3_),^31^ or ammonium persulphate ((NH_4_)_2_S_2_O_8_).^20^ For instance, Youngsoo Kim *et al.* reported a monolayer graphene film with sheet resistance of 923 + 148 Ω sq^−1^ by transferring CVD-grown graphene with PMMA and etching a Cu substrate with 0.02 mol L^−1^ (NH_4_)_2_S_2_O_8_ solutions.^32^ For the same growth and transfer process, other studies revealed diverse results with the sheet resistance of graphene films of 1520 + 67.7 Ω sq^−1^.^33^ Similar phenomena appeared in other etching systems. Li *et al.* removed the Cu substrate using a 0.05 g ml^−1^ Fe(NO_3_)_3_ solution for 12 h and obtained graphene films with a sheet resistance of approximately 2100 Ω sq^−1^.^34^ However, the sheet resistance of graphene films from other reports was 425 Ω sq^−1^.^35^ Moreover, for the FeCl_3_ etching system, two studies reported the sheet resistance of graphene films as 510 Ω sq^−1^ and 725 Ω sq^−1^.^36,37^ After broadly reviewing current reports describing the transfer of graphene films, it is noted that the resultant graphene films have quite different electrical properties.

Obviously, the fabrication of graphene films with unstable and uncertain physical properties would greatly prevent it from being used for practical applications. Thus, it is quite necessary and significantly important to establish a clear relationship between the transfer parameters and the electrical performance. In this work, by systematic study, a standard transfer process for graphene films was successfully established with repeated sheet resistance, which is significantly important for evaluating the quality of CVD-grown graphene films and also for the use of next-generation transparent conducting films in practical applications.

## 2. Experimental

Graphene films were synthesized through the traditional CVD method.^5^ A 25 μm-thick Cu substrate was heated to 1000 °C in H_2_ gas with a flow rate of 100 standard cubic centimeters per minute (sccm). After reaching 1000 °C for 10 minutes, 30 sccm CH_4_ was introduced for 30 minutes to grow graphene on the Cu surface. Finally, the Cu foil with graphene on its surface was cooled to room temperature.

The CVD-grown graphene film on the Cu was transferred to a PET substrate through different approaches, including the PDMS method,^24^ PMMA method,^31^ TRT method^20^ and UV glue method.^23^ For the typical UV glue method, a Cu foil with graphene film on its surface was bonded onto PET film with UV glue (HUITIAN, 3118). Subsequently, the epoxy was cured using a UV curing machine (GLINTSUN, GS-300-2PM). Then, the Cu was etched by different etchants, including ammonium persulphate (NH_4_)_2_S_2_O_8_, iron chloride (FeCl_3_), and iron nitrate (Fe(NO_3_)_3_) solutions. Finally, the graphene/PET film was washed with deionized water several times to remove any etchant residuals and was then dried under an atmospheric environment.

The sheet resistance of graphene on a 5 cm × 5 cm square of PET was measured with a four-point probe (Suzhou Jingge Electronic Co., Ltd., ST2258A) at room temperature. The sheet resistance mapping measurement was carried out with a sheet resistance mapping system (SURAGUS, EddyCus TF map 2525 SR). The morphology of graphene on the PET film was characterized by field emission-scanning electron microscopy (FE-SEM, Hitachi S-4800) and atomic force microscopy (AFM, Dimension 3100). Raman spectra were obtained on graphene films transferred by the PMMA-assisted method using a Renishaw microspectrometer with a laser wavelength of 514 nm. X-ray photoelectron spectroscopy (XPS) measurements were performed using a Kratos Axis Ultra delay-line detector (DLD) spectrometer (Kratos Analytical). The Hall Effect Measurement System (HALL 8800) was used to measure the carrier mobility and carrier density of graphene film with 7200 magnetic strength.

## 3. Results and discussion

**Fig. 1:**
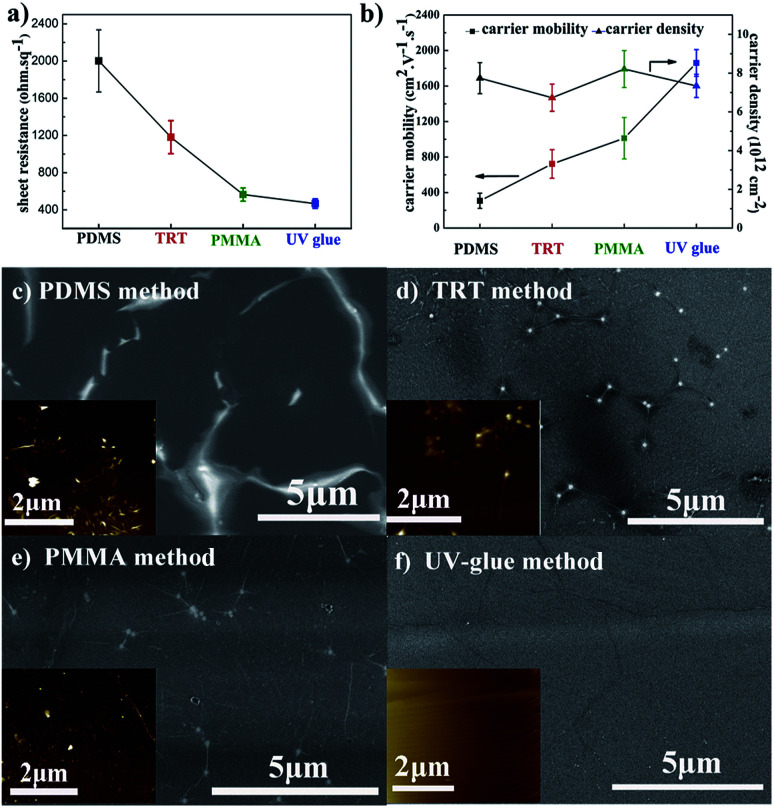
(a) Sheet resistance, (b) carrier density, and carrier mobility of graphene films using different transfer methods. The SEM image and AFM image in the inset of the graphene films transferred by the (c) PDMS method; (d) TRT method; (e) PMMA method; and (f) UV-glue method.

Fig. 1a compares the sheet resistance of graphene films using different transfer methods. The sheet resistance of graphene films is 2001 ± 333 Ω sq^−1^, 1181 ± 178 Ω sq^−1^, 565 ± 70 Ω sq^−1^, and 467 ± 50 Ω sq^−1^, respectively for the PDMS, TRT, PMMA, and UV-glue methods using the same etchant with 0.2 M (NH_4_)_2_S_2_O_8_. It is well-known that the electrical conductivity is primarily determined by two physical parameters: carrier mobility and carrier density. Here, the detailed electrical properties are presented in Fig. 1b. The carrier density of the different samples reveals a similar value of approximately 8 × 10^12^ cm^−2^. It different from the carrier density, the carrier mobility of different samples will exhibit a huge gap. It can be seen that the carrier mobility of graphene transferred by the UV-glue method reaches the highest value of approximately 1800 cm^2^ V^−1^ s^−1^, which is six times higher than that of the PDMS method. These results clearly demonstrate that the large difference in sheet resistance originates from the change in carrier mobility while changing the transfer process.

Because the graphene films on Cu foil are fabricated through exactly the same CVD process, it is quite necessary to investigate the resultant graphene films on PET in detail to further understand their nature. As shown in Fig. 1c–f, the corresponding surface morphologies are characterized by FE-SEM. It should be mentioned that no additional conducting layer was sputtered on the surface in order to ensure the original conditions. Greatly inhomogeneous surfaces with obvious contrast difference can occur, especially in PDMS sample. It is known that poor electrical conductivity during SEM leads to an electrical charge effect.^21,38^ Here, the brighter area might suggest that a poor electrical connection exists, which is due to the cracks that occur during the transfer process. The block of electrical connection would greatly influence the carrier transport and hence decrease the carrier mobility. For comparison, the SEM image of the UV-glue sample is quite uniform on the entire surface with slight wrinkles, which suggests improved carrier mobility. Thus, the final sheet resistance can be significantly different for samples prepared by different transfer methods. AFM measurements were also performed to analyze the microstructures of transferred graphene films, such as ripple or domain boundary,^39,40^ as shown in the insets of Fig. 1c–f. The AFM image of graphene films transferred by the PDMS method exhibits a large oscillation with dense and obvious contrast in some areas. The contrast oscillation becomes much less pronounced in the samples transferred by the TRT and PMMA methods. Finally, it looks quite smooth on the UV-glue assisted transferred graphene film with only slight wrinkling, which is due to the mismatch of the coefficient of thermal expansion between copper and carbon. The results are consistent with the SEM results of graphene films using different transfer methods.

**Fig. 2:**
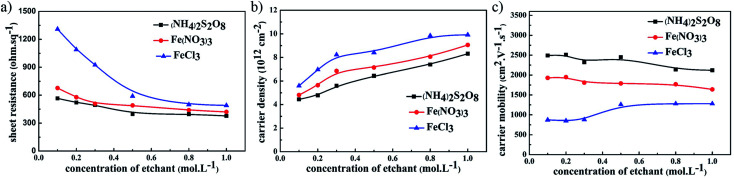
The relationship between (a) sheet resistance, (b) carrier mobility, and (c) carrier density of graphene films and the concentration of different etchants.

The electrical performance of graphene films using different etchants with the same UV-glue transferring method is compared in Fig. 2. For all samples where different etchants were used, including (NH_4_)_2_S_2_O_8_, FeCl_3_, and Fe(NO_3_)_3_ solutions, the sheet resistance of the graphene films gradually decreases as the concentration of etching solutions was increased. Among these three etchants, the graphene films etched in (NH_4_)_2_S_2_O_8_ exhibited the corresponding lowest sheet resistance. The carrier density and carrier mobility of these samples were investigated in detail, and as shown in Fig. 2b, the carrier mobility of graphene films using the same etchant did not greatly change as the etchant concentration increased, and that of graphene films etched in (NH_4_)_2_S_2_O_8_ presents the highest value among these three etchants. In contrast, the carrier density of these graphene films increased significantly as the corresponding etchant concentration increased, as shown in Fig. 2c. Although the carrier density of graphene film etched in (NH_4_)_2_S_2_O_8_ is lower than that of the other samples, the lower sheet resistance is still realized in graphene films etched in (NH_4_)_2_S_2_O_8_, which originates from the large enhancement of carrier mobility. It can be concluded that an increase in ion concentration may not affect the carrier mobility, which remains with an unchanged electrical connection, but greatly enhances the carrier density and hence reduces the sheet resistance.

**Fig. 3:**
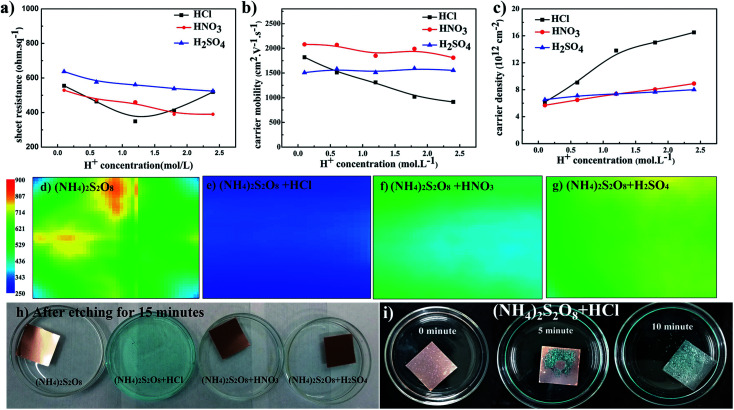
The relationship between (a) sheet resistance, (b) carrier mobility, and (c) carrier density of graphene films and the concentration of different acids in etching solutions; sheet resistance mapping images of 5 cm × 5 cm graphene films on PET substrate using etchants consisting of (d) (NH_4_)_2_S_2_O_8_, (e) (NH_4_)_2_S_2_O_8_ and HCl, (f) (NH_4_)_2_S_2_O_8_ and HNO_3_, and (g) (NH4)_2_S_2_O_8_ and H_2_SO_4_; (h) optical image of graphene films after etching for 15 minutes in different etchants, and (i) view of bottoms of graphene films that were etched in (NH_4_)_2_S_2_O_8_ plus HCl solution for different times.

Based on the above analyses, to further improve the electrical conductivity of graphene films, it is very important to find a method that will increase the carrier density without the loss of carrier mobility. This requirement can be satisfied by introducing dopant during the transfer process, and this has been broadly studied.^20,41,42^ During the above studies, it was found that the pH value decreases as the etching process proceeds. Thus, H^+^ ions as dopant were systemically studied herein to determine if this could further improve graphene films. Fig. 3a–c presents the electrical properties of graphene films etched in (NH_4_)_2_S_2_O_8_ solution plus different acids. The sheet resistance of graphene films generally decreases as the concentration of additional acids increases. For HNO_3_ and H_2_SO_4_ as dopants, the carrier mobility of graphene films remains smooth while the corresponding carrier density increases as the concentration of acids increases, which leads to an overall decrease in sheet resistance. The lowest sheet resistance was realized in (NH_4_)_2_S_2_O_8_ etchant with 1.2 M HCl. With increasing HCl concentration, the sheet resistance sharply increased, and with HNO_3_ and H_2_SO_4_, the carrier mobility of samples in HCl linearly decreased. With careful observation as shown in Fig. 3h, the addition of HCl greatly increased the etching speed compared to the other samples. Additionally, as demonstrated in Fig. 3i, which shows the bottom view of samples during the transfer process, abundant bubbles can be generated as the concentration of HCl increases, which suggests that the violent reaction during the etching process might cause the loss of electrical connections and hence a decrease in carrier mobility. However, the corresponding carrier density sharply increases when HCl is used at concentrations lower than 1.2 M but with a lower slope above 1.2 M. As concluded in the above paragraph, it is necessary to maintain a good electrical connection and hence the carrier mobility. Herein, a suitable HCl concentration could enable the balance between the carrier mobility and carrier density to reach the lowest sheet resistance of approximately 300 ohm sq^−1^. Sheet resistance mapping measurements were also performed to reveal the exact electrical performance of 5 cm × 5 cm graphene films by detecting eddy current signals, as shown in Fig. 3d–g. The sheet resistance mapping of graphene film using only (NH_4_)_2_S_2_O_8_ etchant exhibits complex color units over the entire sample, which indicates the quite disordered distribution of sheet resistance in the range of 350–700 ohm sq^−1^. After adding different acids, the sheet resistance was significantly reduced, and the homogeneity of the sheet resistance was greatly improved with much more uniform color distribution. Among these three types of acids, the sample with the addition of HCl exhibited the lowest sheet resistance, of approximately 300 ohm sq^−1^. These results are consistent with the four-probe measurements and Hall effects measurements.

**Fig. 4:**
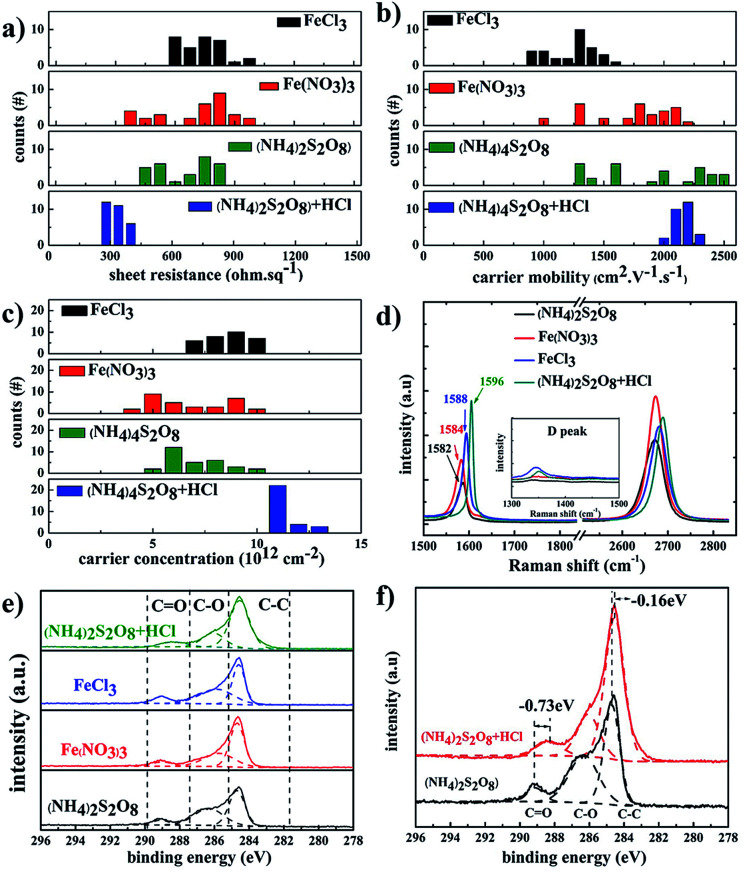
Histogram of (a) sheet resistance, (b) carrier mobility, and (c) carrier density of graphene films etched in different etchants; (d) Raman spectra of graphene films etched in different etchants; (e) XPS peaks of monolayer graphene films transferred to SiO_2_/Si substrates by different etchants; and (f) the XPS peaks of monolayer graphene films etched in (NH_4_)_2_S_2_O_8_ etchant with/without addition of HCl.

Fig. 4 summarizes the electrical properties of graphene films treated with different etchants. The graphene films etched in (NH_4_)_2_S_2_O_8_ etchant exhibited lower sheet resistance compared to that of FeCl_3_ and Fe(NO_3_)_3_ etchants, which is derived from the contribution of higher carrier mobility. With the addition of HCl as a doping agent, the carrier density of samples in (NH_4_)_2_S_2_O_8_ etchant plus HCl becomes more uniform in the range of 2000–2400 cm^2^ V^−1^ s^−1^ compared to a broad range of 1300–2500 cm^2^ V^−1^ s^−1^ for samples in bare (NH_4_)_2_S_2_O_8_ etchant. The corresponding carrier density of samples in the (NH_4_)_2_S_2_O_8_ etchant plus HCl is also significantly improved in a narrow range of 9–12 × 10^12^ cm^−2^ compared to 4–10 × 10^12^ cm^−2^ for samples in bare (NH_4_)_2_S_2_O_8_ etchant. Thus, the sheet resistance of graphene films using HCl dopant reaches the lowest value of approximately 300 ohm sq^−1^ and is also in a much narrower range of 270–380 ohm sq^−1^ compared to the broad range of 350–700 ohm sq^−1^ for the graphene films treated with bare (NH_4_)_2_S_2_O_8_ etchant. This demonstrates that the sheet resistance of graphene films can be repeatedly realized in a quite narrow range using (NH_4_)_2_S_2_O_8_ etchant plus 1.2 M HCl dopant. For the original graphene films, the G peak is at approximately 1582 cm^−1^ and the D band is at approximately 2700 cm^−1^. The doped graphene will exhibit an upshift of the G band and 2D band.^43–46^Fig. 4d presents the Raman spectra of as-obtained graphene films using different etching solutions. It clearly shows that the G band of graphene films etched in Fe(NO_3_)_3_ and FeCl_3_ is slightly upshifted compared to that of the (NH_4_)_2_S_2_O_8_ sample, which is consistent with the carrier density action in Fig. 2c. The higher G band was upshifted to 1596 cm^−1^ in graphene films using (NH_4_)_2_S_2_O_8_ etchant plus 1.2 M HCl dopant, which is also of the highest carrier density and hence lowest sheet resistance.

To further study the doping effect of different etchants, XPS measurements were also carried out, as shown in Fig. 4e and f. The C 1s peaks of the graphene films etched by different etchants are divided into three symmetric Gaussian curves, which are assigned to C–C, C–O, and C=O, respectively. For the p-doping of graphene films, the C 1s peaks corresponding to the sp^2^ and sp^3^ hybridized states were shifted to lower energy,^20,47^ which is similar to the case for p-doped carbon nanotubes.^48^ The peaks at 288.372 eV, 289.049 eV, 289.084 eV, and 289.097 eV correspond to the C=O peaks for graphene films using (NH_4_)_2_S_2_O_8_ etchants plus HCl, FeCl_3_ etchants, Fe(NO_3_)_3_ etchants, and (NH_4_)_2_S_2_O_8_ etchants, respectively. The C=O peak is obviously redshifted by 0.73 eV, and the C–C peak is redshifted by 0.16 eV after the addition of HCl dopant in (NH_4_)_2_S_2_O_8_ etchants, which is consistent with the Raman results.

## 4. Conclusions

In summary, the relationship between the electrical properties of graphene films on PET substrate and the transfer parameters has been systematically investigated in detail. It was found that the guarantee of electrical connections is closely related to the carrier mobility. Additionally, the selection of etchant and dopant is very sensitive to the carrier density and hence influences the sheet resistance. Finally, graphene films can repeatedly reach a low sheet resistance of approximately 300 ohm sq^−1^ with a narrow range if a (NH_4_)_2_S_2_O_8_ etchant with 1.2 M HCl dopant is used. This work provides a facile and reliable technique to obtain graphene films with advanced and repeatable electrical properties, which is critically important for the fair evaluation of CVD-grown graphene films and also the practical application in the field of electrical devices.

## Conflicts of interest

There are no conflicts to declare.

## Supplementary Material
